# Overlap Spectrum Fiber Bragg Grating Sensor Based on Light Power Demodulation

**DOI:** 10.3390/s18051597

**Published:** 2018-05-17

**Authors:** Hao Zhang, Junzhen Jiang, Shuang Liu, Huaixi Chen, Xiaoqian Zheng, Yishen Qiu

**Affiliations:** 1 Department of Electronic Information Science, Fujian Jiangxia University, Fuzhou 350007, China; 1698011400281@fjjxu.edu.cn (S.L.); zxqyx789@163.com (X.Z.); 2 Fujian Provincial Key Laboratory for Photonics Technology, Fujian Normal University, Fuzhou 350007, China; jzjiang@fjnu.edu.cn (J.J.); ysqiu@fjnu.edu.cn (Y.Q.); 3 Fujian Institute of Research on the Structure of Matter, Chinese Academy of Sciences, Fuzhou 350002, China; hxchen@fjirsm.ac.cn

**Keywords:** fiber Bragg grating, demodulation, overlap spectrum, temperature sensors

## Abstract

Demodulation is a bottleneck for applications involving fiber Bragg gratings (FBGs). An overlap spectrum FBG sensor based on a light power demodulation method is presented in this paper. The demodulation method uses two chirp FBGs (cFBGs) of which the reflection spectra partially overlap each other. The light power variation of the overlap spectrum can be linked to changes in the measurand, and the sensor function can be realized via this relationship. A temperature experiment showed that the relationship between the overlap power spectrum of the FBG sensor and temperature had good linearity and agreed with the theoretical analysis.

## 1. Introduction

Fiber Bragg gratings (FBGs) are key passive optical components with numerous advantages including an all-fiber geometry, small size, low insertion loss, good insulation, and high flexibility [1]. As a result, they have attracted considerable attention because of their wide range of applications in fiber sensors, fiber filters, fiber lasers, dispersion compensators, and other optoelectronic devices [2,3,4,5]. Fiber sensors based on FBGs have high sensitivity and resolution and facilitate measurements via changes in reflection spectrum or transmitted spectrum induced by the measured entities [6]. FBGs are widely used to measure temperature, stress, humidity, and many other physical quantities [7,8,9,10].

One of the key issues associated with FBGs sensors that is actively under investigation is signal demodulation. Traditional demodulation methods make use of an optical spectrum analyzer (OSA) [1]. The advantages of this method include a simplified construction and extremely high resolution. However, the OSA is extremely expensive and inconvenient to perform. Thus, this approach is impractical for real-world application. Alternative methods based on well-known optical instruments and tools, such as Fabry-Perot filters, the Mach–Zehnder interferometer, and linear edge filters have been investigated for FBG demodulation to reduce the difficulty and the cost of applications of FBG [11,12,13,14]. However, these methods are limited to some extent by a range of factors including low sensitivity, limited ability to easily adjust the measurement range, high complexity, and the utilization of devices that are not readily available [15].

A novel overlap spectrum FBG sensor based on light power demodulation is presented in this paper. It uses two chirp FBGs (cFBGs) of which the reflection spectra partially overlap each other to realize sensor function. The light power of the overlap spectrum can be associated with changes in a measurand. The design has a simple structure and a low cost. A temperature measurement experiment was designed to test the performance of the FBG sensor. The experimental results revealed that the relationship between the output light power of the FBG sensor and temperature could exhibit good linearity, and the measurement range could be estimated and easily controlled.

## 2. Configuration and Principles

**Figure 1:**
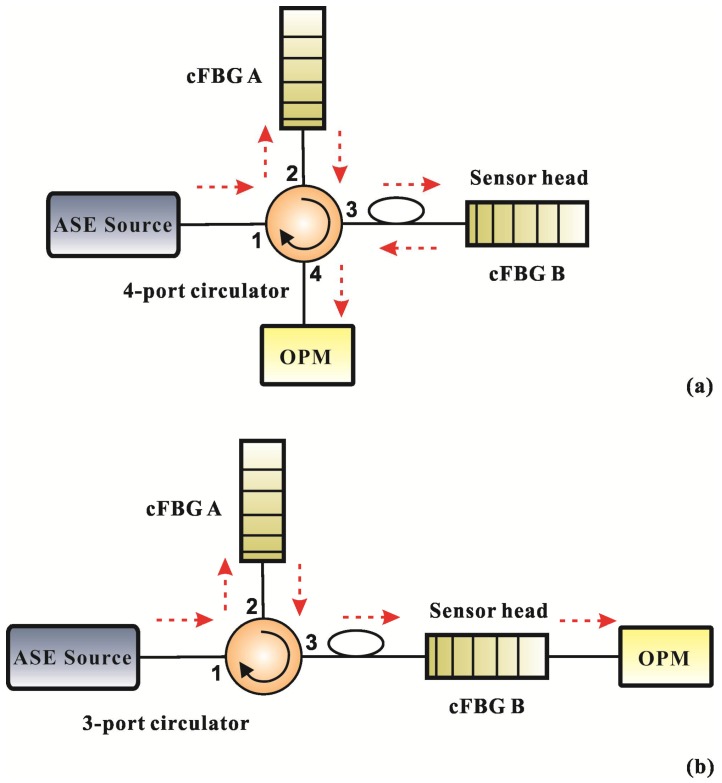
Configuration of an overlap spectrum fiber Bragg gratings (FBG) sensor based on light power demodulation, (**a**) using a four-port circulator, and (**b**) using a three-port circulator.

The two configurations of the overlap spectrum FBG sensor based on light power demodulation are shown in Figure 1. The system is comprised of a broadband-amplified spontaneous-emission (ASE) light source, a three-port or four-port circulator, two cFBGs, and an optical power meter (OPM). The reflection spectra of the two cFBGs must partially overlap each other. The broadband light signal from the ASE light source couples to cFBG A via port 2 of the circulator. cFBG A reflects a portion of light that matches the Bragg reflection condition to cFBG B via port 3 of the circulator. cFBG B reflects a portion of light with wavelengths in the overlap spectrum band of the two cFBGs, and the remaining light is transmitted light. The OPM is connected to port 4 of the circulator to receive the reflected light from cFBG B for the configuration using the four-port circulator. The OPM is connected to cFBG B to receive the transmitted light from cFBG B for the configuration using a three-port circulator.

cFBG A works as a broadband filter in the design and cFBG B is a sensitive element or sensor head. The overlap reflection spectrum of the two cFBGs is invariable when they are not affected by environmental change. However, the overlap spectrum will change when one of the cFBGs is externally perturbed. Therefore, the overlap spectrum can be used to monitor any measurand that is capable of influencing the shift of reflection spectrum of cFBG B.

**Figure 2:**
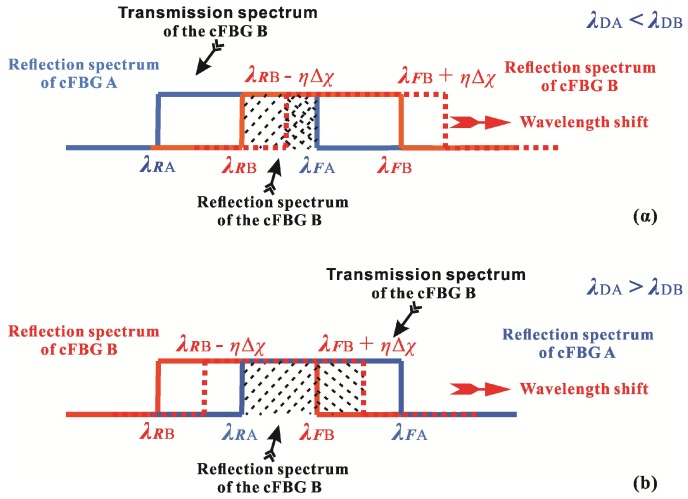
Relationships between the reflection spectra and transmission spectra of the sensor when the wavelength of cFBG B shifts in the long wave direction (**a**) for *λ_DA_* < *λ_DB_*, and (**b**) for *λ_DA_* < *λ_DB_*.

The relationships between the reflection spectra and transmission spectra of cFBGs A and B are shown in Figure 2. For simplicity, the following discussion is based on the wavelength-shift of cFBG B in the long wave direction, and the contrary is the case when the wavelength of cFBG B shifts to the short wave direction. For strong Bragg gratings, the reflected light power of cFBGs A and B can be considered to be concentrated in the range of the full-width-at-half maximum (FWHM) bandwidth, and the reflection spectrum can be treated approximately as a rectangular distribution. The FWHM bandwidths of the reflection spectra of cFBGs A and B are *B_A_* and *B_B_*, respectively, and the edge wavelengths of the reflection spectra of the cFBGs are as follows,

(1) $$\left\{ \begin{array}{l} \lambda_{RA}=\lambda_{DA}-\frac{B_{A}}{2}\\ \lambda_{FA}=\lambda_{DA}+\frac{B_{A}}{2} \end{array}\right.$$

(2) $$\left\{ \begin{array}{l} \lambda_{RB}=\lambda_{DB}-\frac{B_{B}}{2}\\ \lambda_{FB}=\lambda_{DB}+\frac{B_{B}}{2} \end{array}\right.,$$

where *λ_RA_* and *λ_RB_* are the rising-edge wavelengths of the reflection spectra of cFBGs A and B, respectively; *λ_FA_* and *λ_FB_* are the falling-edge wavelengths; and *λ_DA_* and *λ_DB_* are the center wavelengths of cFBGs A and B, respectively. Obviously, the reflection spectrum received by the OPM in Figure 1a is the overlap between cFBGs A and B. The bandwidth of the overlap spectrum is given by:

(3) $$B_{R}=\left\{ \begin{array}{ll} \lambda_{FA}-\lambda_{RB}=\left(\lambda_{DA}+\frac{B_{A}}{2}\right)-\left(\lambda_{DB}-\frac{BB}{2}\right) & \lambda_{DA}<\lambda_{DB}\\ \lambda_{FB}-\lambda_{RA}=\left(\lambda_{DB}+\frac{B_{B}}{2}\right)-\left(\lambda_{DA}-\frac{B_{A}}{2}\right) & \lambda_{DA}>\lambda_{DB} \end{array}\right.$$

Figure 2 shows that the transmitted spectrum from the cFBG B is related to the reflection spectrum. As one increases, and the other decreases necessarily. Therefore, the transmitted light received by the OPM in Figure 1b can also be used to measure the measurand. In theory, the two configurations shown in Figure 1 have the same effect. Furthermore, the bandwidth of transmitted light from the cFBG B in Figure 1b is:

(4) $$B_{T}=\left\{ \begin{array}{ll} \lambda_{RB}-\lambda_{RA}=\left(\lambda_{DB}-\frac{B_{B}}{2}\right)-\left(\lambda_{DA}-\frac{B_{A}}{2}\right) & \lambda_{DA}<\lambda_{DB}\\ \lambda_{FA}-\lambda_{FB}=\left(\lambda_{DA}+\frac{B_{A}}{2}\right)-\left(\lambda_{DB}+\frac{B_{B}}{2}\right) & \lambda_{DA}>\lambda_{DB} \end{array}\right..$$

**Figure 3:**
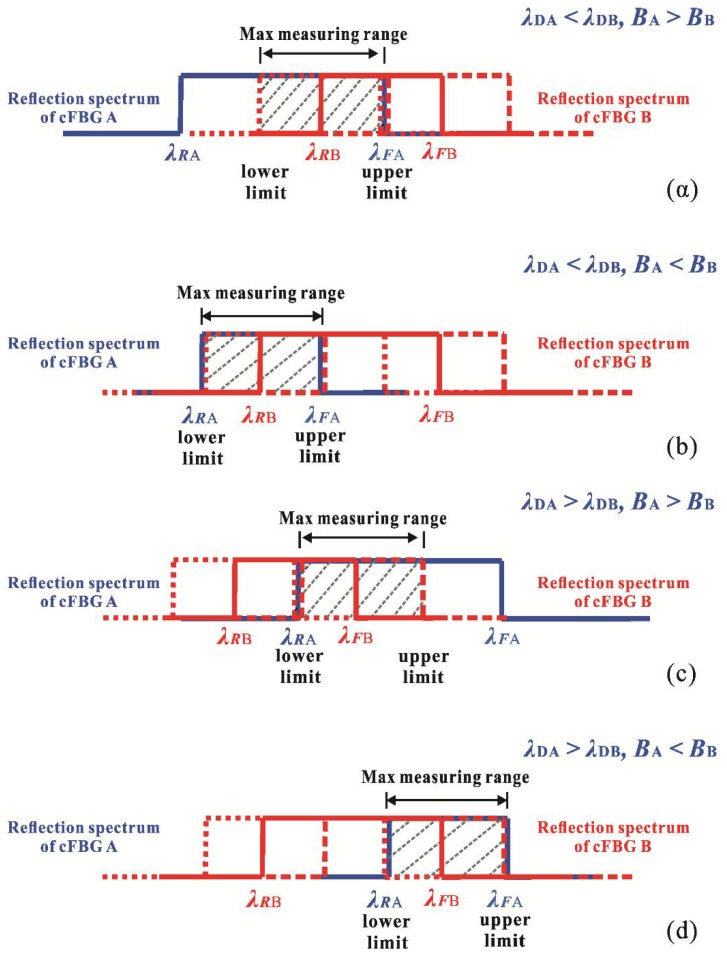
The maximum spectrum range that can be used for measurements when the wavelength of cFBG B shifts to the long wave direction (**a**) for *λ_DA_* < *λ_DB_* and *B_A_* > *B_B_*, (**b**) for *λ_DA_* < *λ_DB_* and *B_A_* < *B_B_*, (**c**) for *λ_DA_* > *λ_DB_* and *B_A_* > *B_B_*, and (**d**) for *λ_DA_* > *λ_DB_* and *B_A_* < *B_B_*.

The reflected light power for the configuration using a four-port circulator can be deduced as:

(5) $$P_{R}=\int_{B_{R}}P(\lambda )d\lambda \approx \frac{B_{R}}{B_{I}}a_{1}P_{I},$$

where *B_I_* is the FWHM bandwidth of input light from the ASE source; *P_I_* is the total power of the input light; and *a*_1_ is attenuation of the system, which is mainly caused by the circulator (port 1 to port 2, port 2 to port 3, and port 3 to port 4). Similarly, the transmitted light power for the configuration using a 3-port circulator is:

(6) $$P_{T}=\int_{B_{T}}P(\lambda )d\lambda \approx \frac{B_{T}}{B_{I}}a_{2}P_{I},$$

where *a*_2_ is the attenuation caused by the circulator (port 1 to port 2, and port 2 to port 3). The Bragg wavelength-shift occurs when cFBG B, which is utilized as a sensitive element, is influenced by a measurand such as temperature, stress, humidity, and so on. The reflected light power and the transmitted light power vary with the Bragg wavelength-shift. The sensitivity coefficient of the wavelength-shift of cFBG B with the measurand is *η*, and the varied reflected and transmitted light power is:

(7) $$\left\{ \begin{array}{l} P_{R}+\Delta P_{R}=\frac{B_{R}\pm \eta \Delta x}{B_{I}}a_{1}P_{I}\\ P_{T}+\Delta P_{T}=\frac{B_{T}\mp \eta \Delta x}{B_{I}}a_{2}P_{I} \end{array}\right.,$$

where Δ*x* is the variation of the measurand. Obviously, the variation of the light power received by the OPM is proportional to Δx, and we have:

(8) $$\left\{ \begin{array}{l} \Delta P_{R}=\frac{\pm \eta a_{1}P_{I}}{B_{I}}\Delta x=\kappa \Delta x\\ \Delta P_{T}=\frac{\mp \eta a_{2}P_{I}}{B_{I}}\Delta x=\kappa \Delta x \end{array}\right.,$$

where κ is the sensitivity coefficient of the overlap spectrum FBG sensor. Figure 3 shows that the light power received by the OPM varies with the wavelength-shift of cFBG B. The wavelength-shift conditions of the upper limit and lower limit of the reflected power and transmitted power are shown in Table 1.

The maximum spectrum range that can be used for measurements is deduced from Figure 3 and Table 1. It is expressed as:

(9) $$R_{\max}=\left\{ \begin{array}{ll} \frac{B_{B}}{\eta } & B_{A}>B_{B}\\ \frac{B_{A}}{\eta } & B_{A}<B_{B} \end{array}\right..$$

Equation (9) shows that the measurement range can be controlled by choosing the FWHM and the sensitivity coefficient *η* of the cFBGs.

## 3. Experiments and Discussion

**Figure 4:**
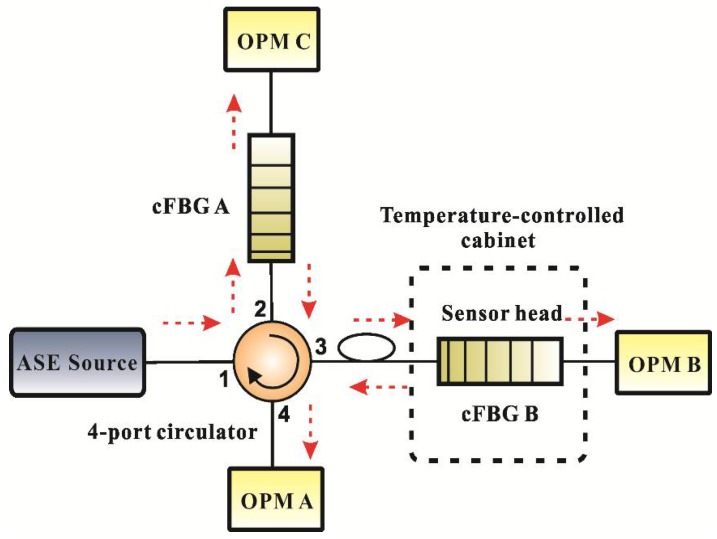
The configuration of a temperature test experiment. See text and Figure 1 for details.

A temperature measurement experiment was conducted to evaluate the performance of the overlap spectrum FBG sensor based on light power demodulation. The experimental layout shown in Figure 4 comprised an ASE light source (Wavephotonics Co., Ltd., Hefei, China, model ASE-30-B; the wavelength covers the C-band; output power 30 mW), a four-port circulator (Shenjian Communication Technology Co., Ltd., Shenzhen, China), two cFBGs, and three OPMs (Shenzhen OSCOM Technology Co., Ltd., Shenzhen, China, model XQ5210). The experimental configuration shown in Figure 4 could receive both reflected and transmitted light from the system. Although both the reflection spectrum and transmitted spectrum have the same effect for measurement as mentioned in theory, there is a gap between theory and reality. Therefore, the experimental configuration shown in Figure 4 combined both configurations shown in Figure 1 and was designed to compare the performance of the two configurations.

**Figure 5:**
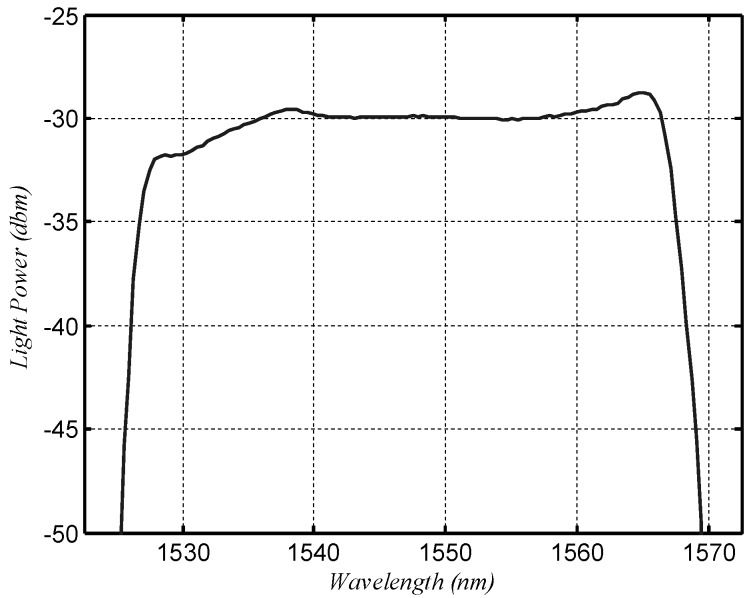
The spectrum of a broadband-amplified spontaneous-emission (ASE) light source.

The spectrum of the ASE source is shown in Figure 5. It shows that the flatness of the spectrum was less than 1 dB from 1537.5 nm to 1562.5 nm. The reflection spectrum of the cFBGs should be in this range to reduce the nonlinearity of the sensor system. cFBG A was fixed at a constant temperature (15 °C), and cFBG B was placed in a temperature-controlled cabinet (Ningbo Scientz Biotechnology Co., Ltd., Ningbo, China, model DL-2020). The temperature was varied from −20 to 100 °C. OPM A was connected to port 4 of the four-port circulator and received reflected light from cFBG B. OPM B was connected to cFBG B and received transmitted light from cFBG B. The accuracy of the measurements can be severely affected by fluctuations of the intensity of the light source. Therefore, the transmitted light from cFBG A received by OPM C was used to monitor the light intensity fluctuations. If the light source fluctuated, the signal detected by OPM A and B were divided by the result received by OPM C in order to suppress the influence of this effect.

**Figure 6:**
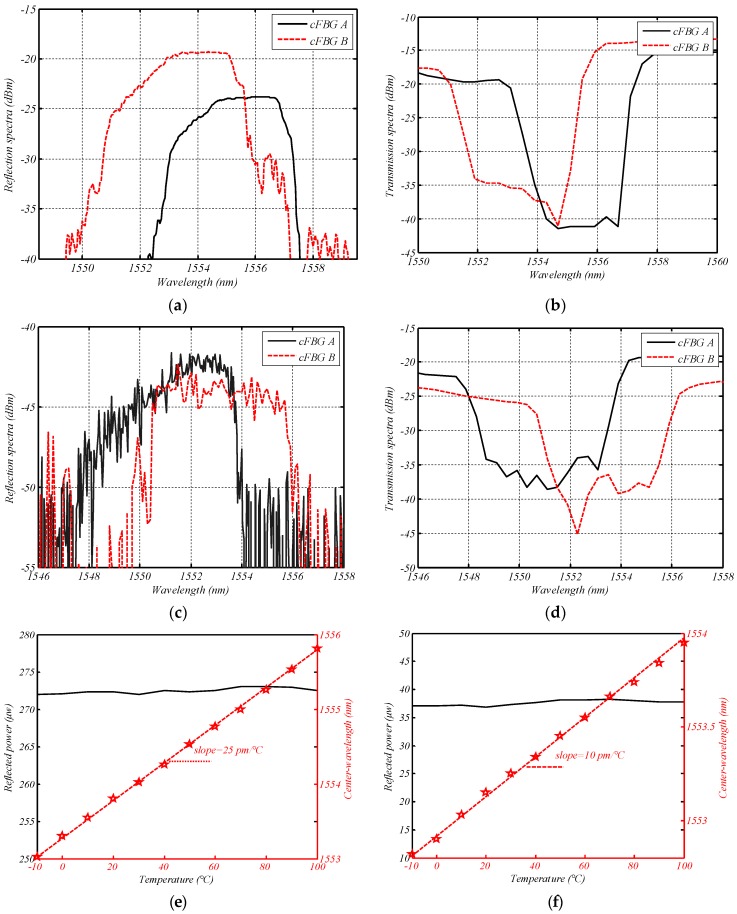
Reflection spectra, transmission spectra, and temperature characteristics of chirp FBGs (cFBGs) at a constant temperature (15 °C) for (**a**) reflection spectra of Group I; (**b**) transmission spectra of Group I; (**c**) reflection spectra of Group II; (**d**) transmission spectra of Group II; (**e**) reflected power vs. temperature, and center-wavelength vs. temperature for the cFBG B used in Group I; and (**f**) reflected power vs. temperature, and center-wavelength vs. temperature for the cFBG B used in Group II.

Two groups of cFBGs were used in the experiment for comparison. Both of their reflection bandwidths were 5 nm, and their reflection spectra were in the flat region of the ASE source. The cFBGs in the first group were apodized, while those in the second group were unapodized. Their reflection and transmission waveforms are shown in Figure 6, and the detailed parameters are shown in Table 2.

Figure 6e presents two relationship curves of the cFBG B of Group I. One is relationship between reflected power and temperature, and the other is center-wavelength vs. temperature. It shows that the reflectivity of cFBG B did not vary with temperature significantly. The slope of the center-wavelength vs. temperature curve is the temperature sensitivity coefficient of cFBG B. Figure 6f shows similar information for the cFBG B of Group II. Obviously, Group I corresponds to the situation in Figure 3c, while Group II corresponds to the situation in Figure 3b. According to Table 1 and Equation (9), the maximum theoretical measurement range of the sensor system was approximately 126 °C for Group I and 377 °C for Group II. The reflection waveform of Group I could be shifted 1.69 nm towards the long wave direction and 1.7 nm towards the short wave direction. This meant that the measurement range for Group I was from 73 °C (15 °C increasing by 58 °C) to −53 °C (15 °C decreasing by 68 °C). Similarly, the theoretical measurement range of Group II was from 342 °C (15 °C increasing by 327 °C) to −35 °C (15 °C decreasing by 50 °C).

**Figure 7:**
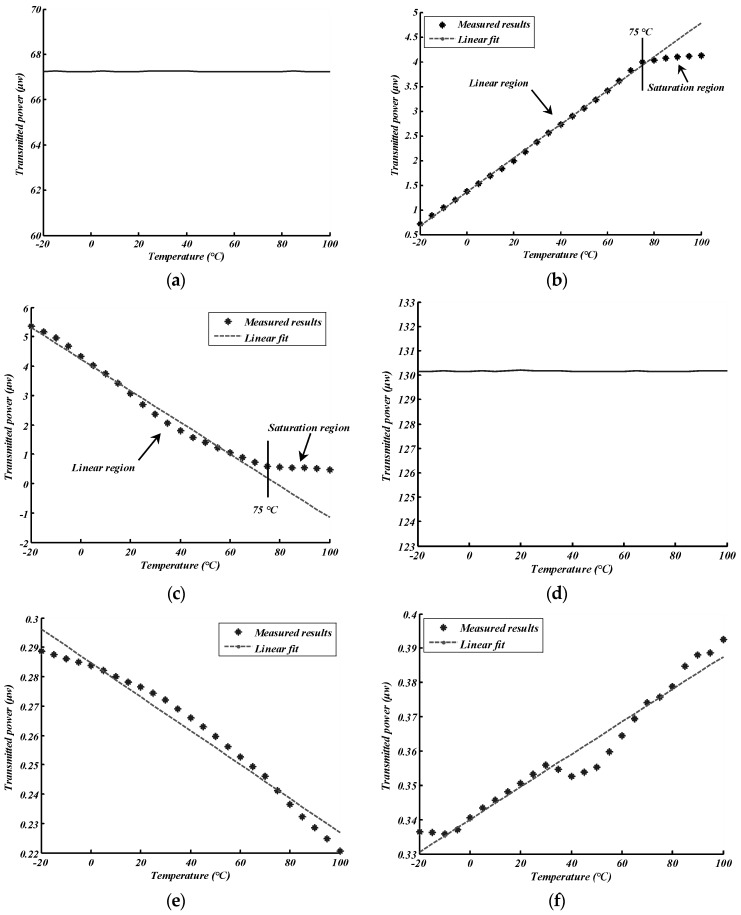
The relationship between light power and temperature (**a**) for transmitted light from cFBG A in Group I, (**b**) for reflected light from cFBG B in Group I, (**c**) for transmitted light from cFBG B in Group I, (**d**) for transmitted light from cFBG A in Group II, (**e**) for reflected light from cFBG B in Group II, and (**f**) for transmitted light from cFBG B in Group II. The dashed line in the figure is a linear fit using the least square method.

The experimental results were recorded at 5 °C intervals and after 20 min, before the temperature reached the set value to uniformly heat the FBG. The relationship between the reflected (transmitted) power and the temperature is shown in Figure 7. Figure 7a–c shows the experimental results for Group I, and Figure 7d–f shows the results for Group II. Figure 7a,d indicates that the light source used in the experiment was fairly stable. Some interesting phenomena and results can be observed in Figure 7.

First, the relationship curves between power and temperature for Group I were more smooth and linear when compared to the results for Group II. This was because the cFBGs in Group I were apodized. Their reflection spectra were therefore smooth, and the sidelobes were reduced.

Second, Figure 7b,c shows that the relationships between the power of the light and temperature for Group I can be divided into two parts: a linear region and a saturation region. The turning point was at 75 °C. The relationship curves had good linearity and the steep slope was in the linear region. However, the slope became flat in the saturation region, as the overlap spectrum had a maximum in the saturation region as shown in Figure 3c. Therefore, this temperature was beyond the measurement range of the system for Group I. According to the analysis of the measurement range, the upper limit of the system for Group I was 15 °C increasing by 58 °C. Thus, the upper limit was 73 °C. This was in exact agreement with the experimental results. The curves in Figure 7e,f did not indicate a significant variation of the slope as the measurement range of the system in the case of Group II was large. The temperature range in the experiment did not extend beyond the measurement range.

Finally, although using both the reflection spectrum for measurement and using the transmitted spectrum for measurement had the same performance in theory, the experimental results revealed different phenomena. The sensitivity when using the reflection spectrum in Group I was 0.034 μw/°C, and −0.054 μw/°C when using the transmitted spectrum. In Group II, the sensitivity was −0.578 nw/°C when using the reflection spectrum, and 0.425 nw/°C when using the transmitted spectrum. Furthermore, the linearity of the reflected power vs. temperature curve was better than that of the transmitted power vs. temperature curve. For example, the non-linear error of the linear region in Figure 7b was ~1.75%, and ~7.77% in Figure 7c. Similarly, the non-linear error was ~10.84% for Figure 7e and ~14.75% for Figure 7f. An important reason for the difference is that the reflection spectrum of FBG was not a perfect rectangular distribution and there were some sidelobes in the reflection spectrum of FBG. The overlap reflection spectrum from cFBG B reduced more sidelobes than the transmitted spectrum because it was reflected twice by cFBG A and cFBG B. Therefore, a sensor system using reflected light from cFBG B is expected to perform better than a system using transmitted light.

## 4. Conclusions

In summary, an overlap spectrum FBG sensor based on light power demodulation was presented. The sensor used the overlap spectrum power of two cFBGs to detect changes in a measurand. Both the theoretical analysis and experimental results indicated that the relationship between the overlap spectrum power and the measurand exhibited good linearity. The experiment also revealed that FBG apodization and the configuration using reflected light could improve the performance of the sensor.

## Figures and Tables

**Table 1:** Wavelength-shift condition of the upper limit and lower limit of the reflected power.

Light Power	Center Wavelength	FWHM Condition	Wavelength-Shift Condition
Upper limit (reflected)/lower limit (transmitted)	*λ_DA_* *< λ_DB_*	*B_A_* *> B_B_*	*λ_FB_ = λ_FA_*
	*λ_DA_* *< λ_DB_*	*B_A_* *< B_B_*	*λ_RB_ = λ_RA_*
	*λ_DA_* *> λ_DB_*	*B_A_* *> B_B_*	*λ_RB_ = λ_RA_*
	*λ_DA_* *> λ_DB_*	*B_A_* *< B_B_*	*λ_FB_ = λ_FA_*
Lower limit (reflected)/upper limit (reflected)	*λ_DA_* *< λ_DB_*	*B_A_* *> B_B_*	*λ_RB_ = λ_FA_*
	*λ_DA_* *< λ_DB_*	*B_A_* *< B_B_*	*λ_RB_ = λ_FA_*
	*λ_DA_* *> λ_DB_*	*B_A_* *> B_B_*	*λ_FB_ = λ_RA_*
	*λ_DA_* *> λ_DB_*	*B_A_* *< B_B_*	*λ_FB_ = λ_RA_*

Note: FWHM denotes the full-width-at-half maximum bandwidth.

**Table 2:** The detailed parameters of the cFBGs used in the experiment.

Group	Identifier	Center-Wavelength (nm, 15 °C)	Rising-Edge Wavelength (nm)	Falling-Edge Wavelength (nm)	FWHM (nm)	Temperature Sensitivity Coefficient (pm/°C)
I	A	1555.34	1553.65	1557.04	3.39	10
	B	1553.67	1552.19	1555.35	3.16	25
II	A	1551.37	1550.00	1553.77	3.77	10
	B	1553.09	1550.50	1555.73	5.23	10
